# A Rare Case of Erythema Gyratum Repens Associated With Esophageal Carcinoma

**DOI:** 10.7759/cureus.9971

**Published:** 2020-08-23

**Authors:** Abhishek Matta

**Affiliations:** 1 Internal Medicine, University of North Dakota, Fargo, USA; 2 Internal Medicine, Sanford Health, Fargo, USA

**Keywords:** erythema gyratum repens, malignancy associated rash, serpiginous rash, esophageal carcinoma

## Abstract

Rash is a common complaint in a primary care setting. Erythema gyratum repens (EGR) is a unique rash strongly associated with malignancy. Sometimes this rash can precede the clinical presentation of malignancy, most commonly lung carcinoma. Even though this is an uncommon rash, physicians need to be aware of this condition for the prompt evaluation of malignancy to start the therapy. In this report we present the case of a 61-year-old gentleman with stage IV squamous cell carcinoma of the esophagus who presented with EGR two months after the diagnosis of his malignancy. The diagnosis was made based on clinical exam and histological findings. The patient was reassured and the rash was managed conservatively. Chemotherapy was continued and the rash was resolved in two months.

## Introduction

Erythema gyratum repens (EGR) is a distinctive rash often indicative of an underlying internal malignancy. However, recent reports showed 30% of the time it is idiopathic [1]. EGR is associated with a variety of genitourinary, gastrointestinal, and hematological malignancies notably bronchogenic, esophageal, and breast cancer [2-4]. It is also seen in patients with non-neoplastic conditions such as tuberculosis, hypereosinophilic syndrome, pregnancy, calcinosis, CREST syndrome, bullous pemphigoid, pemphigus vulgaris, systemic lupus erythematosus, ulcerative colitis, and rheumatoid arthritis [2, 5-7]. EGR was first described by Gammel [8] in 1952 in a patient nine months before the appearance of breast cancer. The average age of onset is 63 years with male predominance. Most of the reported cases are Caucasians [2].

## Case presentation

**Figure 1:**
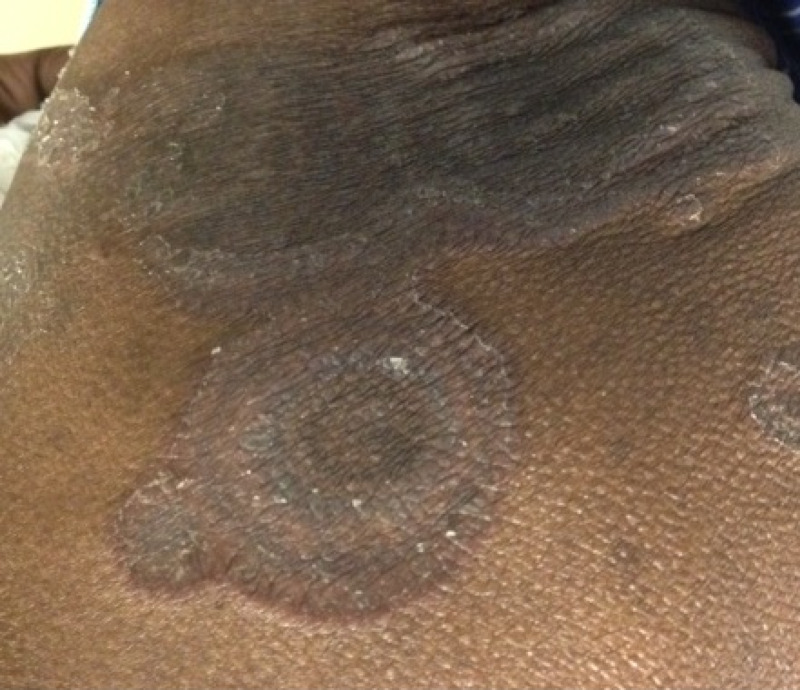
Erythema gyratum repens.

**Figure 2:**
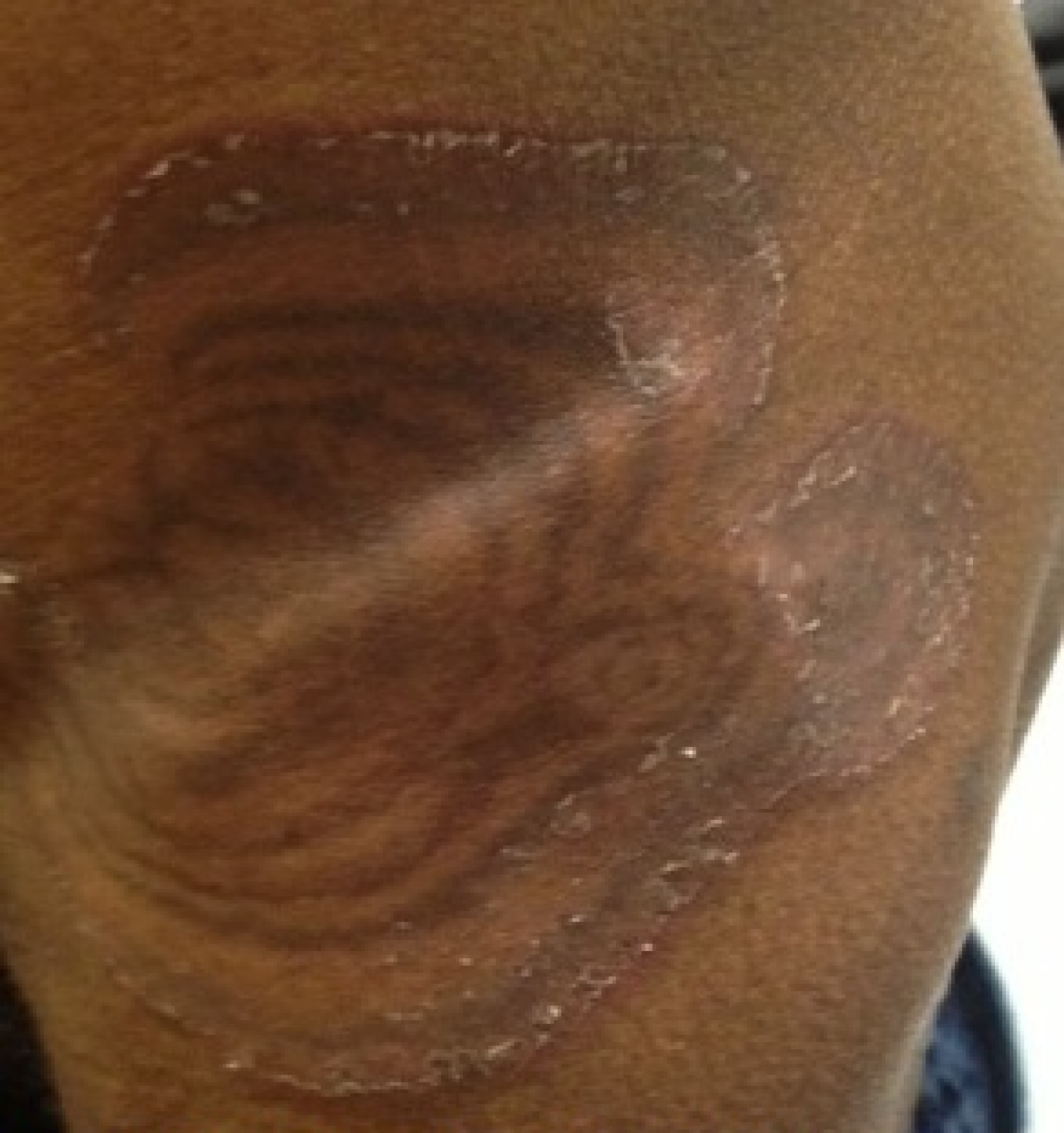
Erythema gyratum repens.

**Figure 3:**
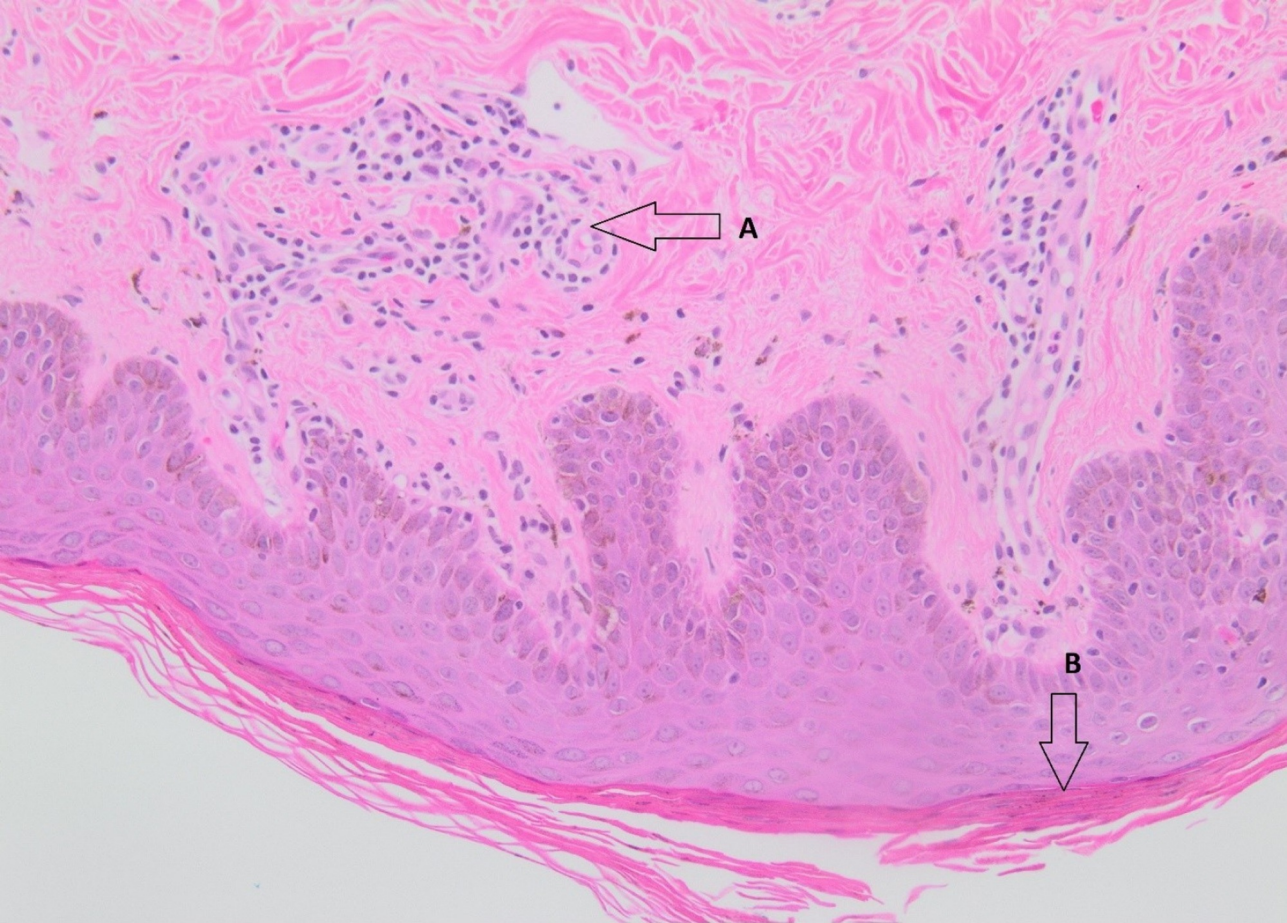
Histology of erythema gyratum repens. A: Perivascular lymphocytic infiltration. B. Parakeratosis.

A 61-year-old African-American gentleman with no significant medical history presented to our clinic with dysphagia and progressive weight loss. He had a history of tobacco abuse. He was hemodynamically stable and physical examination was unremarkable. Esophagogastroduodenoscopy revealed esophageal carcinoma and CT abdomen revealed metastasis to the pancreas. He was started on chemotherapy with carboplatin and paclitaxel. Two months later he developed a rapidly progressive pruritic rash on his left thigh that progressively worsened to involve most of the trunk. On examination, distinctive serpiginous scaling patches with wood-grained appearance were noted on the thighs and trunk (Figures 1-2). A clinical diagnosis of EGR was made. A five-millimeter punch biopsy was obtained from the lateral thigh. Histology showed mild chronic perivascular lymphocytic infiltration with pigmented macrophages, epidermal parakeratosis, and focal spongiosis (Figure 3). The patient was educated about EGR and reassured. Chemotherapy was continued and the rash resolved in two months.

## Discussion

Erythema gyratum repens is a rash that is predominantly associated with malignancy. A systematic review reported that EGR was associated with underlying malignancy in 70% of the cases [1]. The exact pathogenesis is unknown but postulated to be secondary to an immune response triggered by the underlying malignancy [1].

Clinically the lesions are serpiginous, macular (occasionally papular), and erythematous with desquamating edges. Numerous serpiginous figures give rise to a characteristic “wood-grained” appearance [9]. These lesions can extend rapidly, estimated at 1 cm/d in some patients. The hands, feet, and face are commonly spared [2, 4]. Occasional features include volar hyperkeratosis, ichthyosis, bullae, and onychodystrophy [4, 10]. Pruritus is almost always seen.

Differential diagnosis includes erythema annulare centrifugum (EAC), necrolytic migratory erythema (NME), and erythema migrans. Diagnosis is usually made clinically. Histopathology is nonspecific, often showing mild hyperkeratosis, parakeratosis, acanthosis, and spongiosis with a perivascular mononuclear inflammatory infiltrate in the dermis [11-12]. The observed immunofluorescence patterns of immunoglobulin G, C3, and C4 at the basement membrane corroborate a possible immunologic mechanism [4]. Eosinophilia is observed in approximately 60% of cases.

There is no specific therapy for the EGR. Topical and systemic steroids, vitamin A, and azathioprine have proven to be not effective [2, 4, 13]. If a patient presents with the characteristic rash, an underlying malignancy should be suspected and investigated. Recognition and treatment of the underlying condition usually but not always lead to resolution of the rash [4].

## Conclusions

Erythema gyratum repens is a rare distinctive rash associated predominantly with underlying malignancy. The diagnosis is made clinically. Identification of the rash should prompt investigation into underlying conditions that can trigger the rash. Addressing the underlying condition will lead to improvement of the rash.
